# Ratiometric Sensing of Glyphosate in Water Using Dual Fluorescent Carbon Dots

**DOI:** 10.3390/s23115200

**Published:** 2023-05-30

**Authors:** Adryanne Clermont-Paquette, Diego-Andrés Mendoza, Amir Sadeghi, Alisa Piekny, Rafik Naccache

**Affiliations:** 1 Center for NanoScience Research, Department of Chemistry and Biochemistry, Concordia University, Montreal, QC H4B 1R6, Canada; 2 Quebec Centre for Advanced Materials, Department of Chemistry and Biochemistry, Concordia University, Montreal, QC H4B 1R6, Canada; 3 Centre for Microscopy and Cellular Imaging, Department of Biology, Concordia University, Montreal, QC H4B 1R6, Canada

**Keywords:** carbon dots, sensing, glyphosate, ratiometric fluorescence

## Abstract

Glyphosate is a broad-spectrum pesticide used in crops and is found in many products used by industry and consumers. Unfortunately, glyphosate has been shown to have some toxicity toward many organisms found in our ecosystems and has been reported to have carcinogenic effects on humans. Hence, there is a need to develop novel nanosensors that are more sensitive and facile and permit rapid detection. Current optical-based assays are limited as they rely on changes in signal intensity, which can be affected by multiple factors in the sample. Herein, we report the development of a dual emissive carbon dot (CD) system that can be used to optically detect glyphosate pesticides in water at different pH levels. The fluorescent CDs emit blue and red fluorescence, which we exploit as a ratiometric self-referencing assay. We observe red fluorescence quenching with increasing concentrations of glyphosate in the solution, ascribed to the interaction of the glyphosate pesticide with the CD surface. The blue fluorescence remains unaffected and serves as a reference in this ratiometric approach. Using fluorescence quenching assays, a ratiometric response is observed in the ppm range with detection limits as low as 0.03 ppm. Our CDs can be used to detect other pesticides and contaminants in water, as cost-effective and simple environmental nanosensors.

## 1. Introduction

Nowadays, pesticides and herbicides are widely used in agriculture to improve the quality and yield of crops. Glyphosate (*N*-(phosphonomethyl)glycine) is a broad-spectrum, non-selective pesticide used for crops, vegetation, and weed control. It is currently globally used and is found in more than 750 products for agriculture, forestry, urban, and home use. Because of its relatively low toxicity toward mammals, glyphosate has become the most widely used pesticide; however, recent studies revealed that the overuse of glyphosate is directly linked to environmental pollution [1,2,3]. Glyphosate has strong soil retention and high solubility in water, as well as a long half-life in the environment [4,5]. According to the International Agency for Research on Cancer (IARC), this herbicide has been linked to possible carcinogenic effects in humans [6]. As a consequence, glyphosate represents a health hazard, highlighting the urgent need to monitor its presence in the environment.

To date, chromatography techniques such as high-performance liquid chromatography and gas chromatography remain the most popular approaches to evaluate the concentrations of glyphosate in samples. Capillary electrophoresis and inductively coupled plasma–mass spectrometry have also been established as alternative approaches [7,8,9,10]. While these analytical methods offer high sensitivity and selectivity, they are not cost-effective and require sophisticated instrumentation [11,12]. To overcome these limitations, a novel approach to detect glyphosate relies on fluorescence-based sensors from quantum dots (QDs), organic dyes, metal–organic frameworks, or composites, among many others [13,14,15]. However, the fabrication of these materials poses numerous challenges, such as the requirement for the use of heavy metals that can degrade and leach into the environment, complex syntheses, as well as limited photo- or physical stability. In this context, carbon dots (CDs) are attractive due to their sustainable, cost-effective synthesis and versatile physico-chemical and optical properties [16]. CDs are defined as quasi-spherical carbon-based nanomaterials with a size of ~10 nm [17]. They can be synthesized using facile, accessible, and low-cost synthesis routes. They possess unique properties, such as tunable emission, high fluorescence quantum yields, excellent water dispersibility, low cytotoxicity, and good biocompatibility. Moreover, their surfaces are rich in functional groups, enabling chemical tunability and reactivity with the surrounding environment. For this reason, they have been exploited for a range of applications in the fields of sensing, energy storage, catalysis, and theragnostics [18,19,20].

In recent years, several CD-based fluorescent sensors have been developed to detect pesticides and have shown the potential to compete with the currently used analytical techniques (Table 1). Hou et al. used a hydrothermal method to synthesize CDs from Sophora japonica leaves, where fluorescence is quenched by the addition of Fe^3+^ and recovers by interaction with glyphosate [21]. 

This probe detects glyphosate ranging from 0.1 to 16 ppm, with a detection limit of 0.008 ppm. Wu et al. designed green emissive carbon dots with similar properties, which are quenched by the addition of Cu^2+^ ions, and fluorescence is recovered by glyphosate up to 110%, with a detection range of 0.5–1.3 ppm [29]. While fluorescence quenching/recovery offer improved sensitivity and selectivity, as well as portability and a rapid response when compared to other techniques, they are limited by environmental factors and the instrumentation. Ratiometric sensing overcomes these challenges and offers improved sensitivity [30,31,32]. Indeed, by assessing changes in the ratio between two fluorescent signals as a function of the concentration of the target analyte, precise measurements can be obtained regardless of the external environmental conditions or fluctuations and artefacts associated with instrumentation [33]. Only a few ratiometric carbon-based sensors have been developed to date. For example, Luo et al. synthesized an N-doped CD encapsulated by a porphyrin metal–organic framework to ratiometrically detect glyphosate with a limit of detection (LOD) of 1.6 ppm, with an integrated smartphone-assisted platform [34,35,36,37].

Herein, we report the microwave-assisted synthesis of dual-emitting CDs from L-glutathione and formamide for the simple and sensitive detection of glyphosate at sub-ppm levels in aqueous solutions with different pH (3–10) to mimic various environmental conditions. Our detection method is based on the ratiometric response of red to blue fluorescence signals following the addition of glyphosate. Glyphosate quantitation assays are developed over low (0–20 ppm) and high (0–500 ppm) concentration ranges and the optical response of the CDs is evaluated over their entire range of emission (400–800 nm). The surface chemistry of the CDs reveals their mechanism of glyphosate interaction. Our CDs demonstrate the competitive, sensitive, and selective detection of glyphosate, with an LOD as low as 0.03 ppm.

## 2. Materials and Methods

### 2.1. Chemicals and Reagents

Formamide (≥99.5%) and reduced L-glutathione (≥98.0%) were purchased from Thermo Scientific. Glyphosate (*N*-(phosphonomethyl)glycine) was purchased from Sigma Aldrich. All reagents and chemicals were used without any further modification or purification.

### 2.2. Synthesis of the CDs

The CDs were prepared in a microwave reactor using a one-step reaction with L-glutathione and formamide, as previously reported [17,31,32]. The CDs were synthesized using a CEM Discover SP Microwave Reactor. A 10 mL solution of L-glutathione (0.1 M) in formamide was prepared and then sonicated for 20 min until the solution turned clear. Subsequently, the solution was transferred to a 35 mL microwave vial and heated to 180 °C for 5 min at 300 psi in the microwave reactor. After the reaction was completed, the solution was dialyzed in Milli-Q water using a cellulose ester dialysis membrane (molecular weight cutoff: 3.5–5.0 kDa) to remove unwanted material and impurities. The water was changed twice a day for seven consecutive days. Afterwards, the solution was dried using the rotavapor until a fine powder was acquired. Lastly, the CDs were purified and washed twice with acetone and twice with ethanol (1:10 volume ratio of sample:solvent). After each wash, the precipitate was collected by centrifugation at 10,000× *g* for 10 min, from which the supernatant was discarded. Finally, the pellet collected was placed in an oven at 80 °C overnight to dry.

### 2.3. Fluorescence Spectroscopy

Fluorescence spectra were measured using a Cary Eclipse fluorescence spectrophotometer from Agilent Technologies. Spectra were acquired in a 10 mm quartz cuvette at λ_ex_ = 405 nm from 200 nm to 800 nm (1 nm intervals). The excitation and emission slits were set to a width of 5 nm, with a PMT voltage of 600 V and a scan rate of 600 nm/min. All data were processed using the Cary Eclipse software. The spectra were background-corrected for the solvent (Milli-Q water).

### 2.4. UV–Visible Absorbance Spectroscopy (UV–Vis)

UV–visible absorption spectra were acquired from 200 to 800 nm on a Cary 5 Series UV–Vis–NIR Spectrophotometer from Agilent Technologies using a 10 mm quartz cuvette. A 5.0 nm bandwidth and wavelength changeover at 350 nm were used for analysis. Data were processed using the Cary Eclipse software. The spectra were background-corrected for the solvent (Milli-Q water).

### 2.5. Fourier Transform Infrared Spectroscopy (FT-IR)

FTIR spectra were collected using a Thermo Scientific Nicolet iS5 equipped with an iD5 ATR accessory. Approximately 10 µg of sample was used for the analysis. The spectra were collected using 64 scans with a resolution of 0.4 cm^−1^, a gain of 1, an optical velocity 0.4747, and an aperture setting of 100. Data were processed using the Omnic 9 software (v.9.2; Thermo Scientific).

### 2.6. X-ray Photoelectron Spectroscopy (XPS)

XPS spectra of the CDs were acquired using a Thermo Scientific K-Alpha X-Ray Photoelectron Spectrometer (ThermoFisher Scientific, Boston, MA, USA). Each analysis was carried out in triplicate, with 10 runs for each scan; the high-resolution and survey scans represent the average of the triplicate measurements. 

### 2.7. Fluorescence Lifetimes

Fluorescence lifetimes were acquired using an EasyLife X fluorescence lifetime system (Optical Building Blocks Corporation, Birmingham, NJ, USA). Spectra were collected in a 1 cm quartz fluorescence cuvette, using a 368 nm pulsed picosecond LED excitation source. An emission slit width of 1.5 mm, 800 channels, 1.0 s integration time, and 1 reading were used for analysis. A bi-exponential decay was measured with a random collection mode in logarithmic collection steps to account for any potential photobleaching and to obtain more data points at the time of the pulse. The data were processed and analyzed using the OBB EasyLife X software (v.10.0.0.38; Optical Building Blocks Corporate, Birmingham, NJ, USA).

### 2.8. Transmission Electron Spectroscopy (TEM)

TEM grids (300-mesh CU (Cu-300HD)) were prepared by pipetting 2 mL of a 2 mg/mL dispersion of CDs onto the surface, followed by the evaporation of the solvent. The TEM images were collected using a JEOL JEM-2100F microscope operating at 100 kV. The images were processed, and carbon dot sizes were measured using the Fiji imaging software (v.1.53; NIH, Bethesda, MD, USA).

## 3. Results and Discussion

### 3.1. Physico-Chemical and Optical Characterization

**Figure 1:**
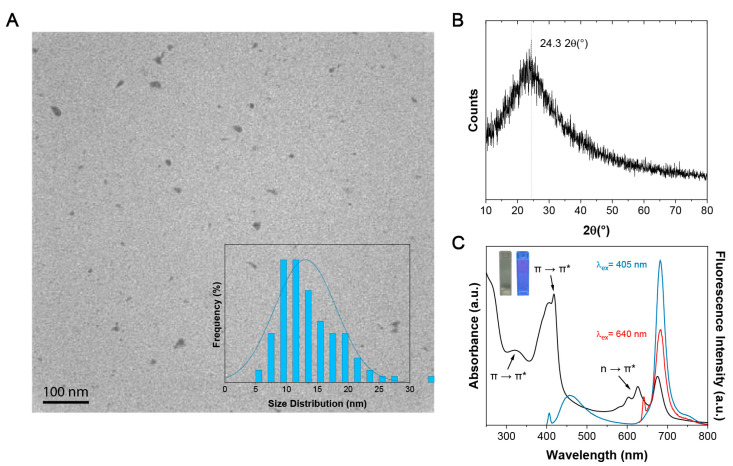
**Physico-chemical properties of CDs.** (**A**) TEM image of a 2 mg/mL CD dispersion in water. A Gaussian size distribution profile ranging from 0 to 30 nm is observed. The average size of the NPs was calculated to be 13.1 ± 5.0 nm (number of counts = 100). (**B**) XRD profile of the CDs shows an amorphous halo in the range of 10–80 2θ, noting the broad graphitic reflection on 24.3 o2q. (**C**) The UV–vis absorption spectrum highlights three prominent bands at 250, 420, and 580–690 nm. At an excitation wavelength of 405 nm, there are two fluorescence bands observed in the blue and red regions of the spectrum, while excitation at 640 nm shows only red fluorescence. Under 365 nm UV light, the CD solution displays a violet-blue color.

CDs were synthesized via a microwave-assisted technique using formamide and L-glutathione as precursors. Following purification and isolation of the dried product, TEM analysis was performed to evaluate the shape and morphology of the dots. In Figure 1A, the TEM digital image reveals that the dots were quasi-spherical with a mean size of 13.1 ± 5.0 nm. Particle size distribution analysis revealed that the dots ranged from 5 to 30 nm in size. The larger sizes were attributed to agglomerations of the dots on the TEM grid. The CDs lacked a crystalline structure, as evidenced by the XRD profile (Figure 1B), with a broad amorphous halo spanning the range of 10–80° 2θ. The broad reflection at 24.3 °2q on top of the amorphous halo was ascribed to the graphitic structure of the dots.

The CDs formed a near-colorless light green solution when dispersed in water at a concentration of 50 μg/mL. When exposed to a wavelength of 365 nm, a violet color was observed, which could be attributed to the simultaneous emission of blue and red fluorescence. The UV–vis absorption spectra of the CDs are shown in Figure 1C. Three spectral bands were observed: the first band was a π → π* transition at 250 nm, ascribed to the C=C bonds; the second band, at 420 nm, was assigned to the π → π* transition of C=O/C=N bonds, while the third band, spanning 580 to 690 nm, was associated with the n → π* transitions of C=S bonds [14,17,31,32]. Upon excitation of the CDs with a short wavelength of 405 nm, dual fluorescence was produced in the blue and red regions of the spectrum, while excitation with a long wavelength of 600 nm produced a single peak centered at 680 nm. This optical effect can be attributed to the core and molecular states of the CDs [38].

**Figure 2:**
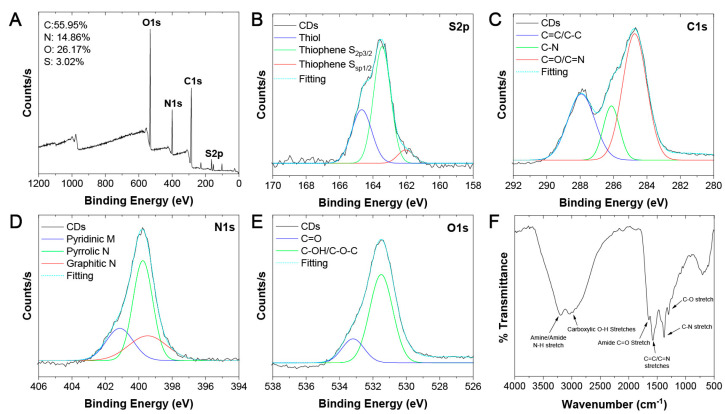
**Chemical composition of dual emissive CDs.** (**A**) XPS survey spectrum of the CDs, which reveals four binding energies for S_2p_, C1s, N1s, and O1s. HR-XPS spectra of the deconvoluted binding energies are assigned to (**B**) S_2p_ at a maximum of 163.4 eV, (**C**) C1s at a maximum of 286.6 eV, (**D**) N1s at a maximum of 400.5 eV, and (**E**) O1s at a maximum of 513.4 eV. (**F**) FT-IR spectrum of the CDs shows the presence of O-H and N-H surface groups in addition to carbonyl and amide stretches.

XPS analysis was performed to explore the chemical composition of the CDs. The survey spectrum in Figure 2A illustrates four binding energies at 533.2, 399.7, 285.1, and 164.9 eV, corresponding to oxygen (O1s), nitrogen (N1s), carbon (C1s), and sulfur (S2s), respectively. The high-resolution XPS (HR-XPS) spectra shown in Figure 2B reveal the binding energies of thiophene S_2p1/2_ (162.1 eV), thiophene S_2p3/2_ (163.4 eV), and thiolate (164.7 eV). In a similar manner, C-C/C=C, C-N, and C=O/C=N functional groups were found by deconvoluting C1s into binding energies of 284.7, 286.1, and 287.9 eV, respectively (Figure 2C). Likewise, deconvolution of the HR-XPS N1s spectrum (Figure 2D) resulted in binding energies of 399.5, 399.8, and 401.2 eV, associated with pyridinic, NH_2_/pyrrolic, and graphitic nitrogen, respectively. Lastly, the HR-XPS O1s spectra (Figure 2E) showed deconvoluted binding energies at 531.5 and 533.2 eV, which could be ascribed to the C-OH/C-O-C and C=O functional groups. The elemental composition of the CDs was measured through XPS analysis by comparing the integrated peak areas in the HR-XPS spectra of the respective elements. The composition of the CDs was found to be 55.95% carbon, 14.86% nitrogen, 26.17% oxygen, and 3.02% sulfur. This composition is similar to that of other glutathione-based CDs discussed in the literature [17,30,32,38].

To further explore the surface properties of the CDs, Fourier transform infrared spectroscopy (FT-IR) was performed, as shown in Figure 2F. The FT-IR spectrum revealed a broad band centered at ~3100 cm^−1^, attributed to O-H and N-H stretching vibrations of hydroxyl and amine groups. Furthermore, three strong bands at 1649, 1375, and 1300 cm^−1^ confirmed the presence of C=O, C-N, and C-O groups, respectively. Lastly, the band appearing at 1575 cm^−1^ was associated with the C=C/C=N groups present in the cores and/or surfaces of the dots. These findings are indeed in accordance with our XPS analysis.

### 3.2. Detection of Glyphosate Using a Ratiometric Approach

**Figure 3:**
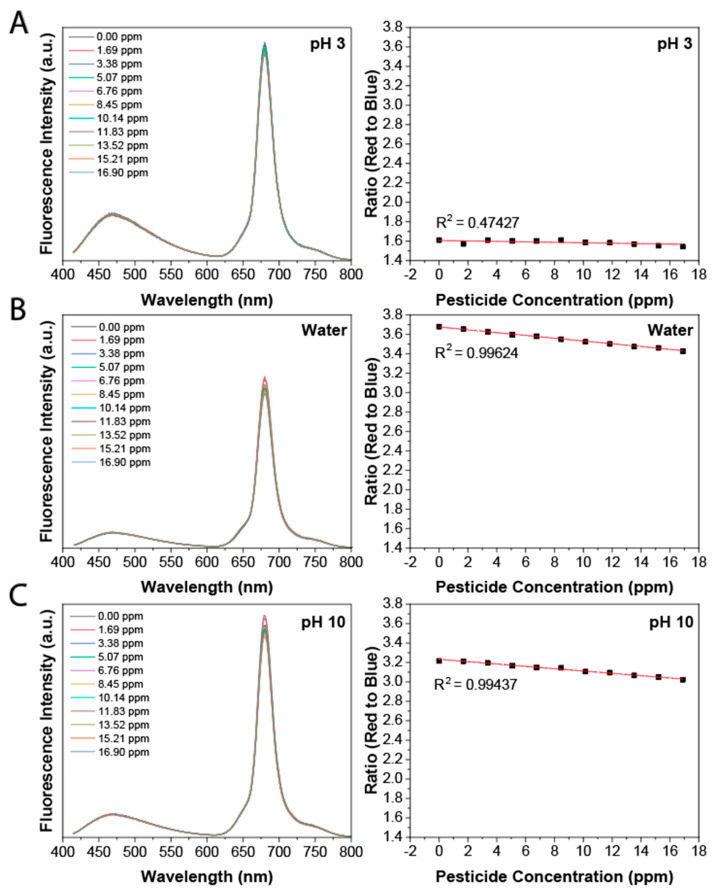
**Low concentration of glyphosate sensing based on ratiometric measurements**. Following excitation at 405 nm, a change in fluorescence is observed in the red fluorescence; the ratio of the integrated area of the red and blue emission peaks shows a negative exponential response to the change in glyphosate concentration. (**A**) At pH 3 with LOD = 9.0 ppm; (**B**) water with LOD = 0.6 ppm; (**C**) at pH 10 with LOD = 5.4 ppm.

Next, we determined the effect of adding glyphosate on the optical properties of the dual emissive CDs. The optical properties of the CDs in water were measured as a function of the glyphosate concentration. As shown in both Figure 3 and Figure S1, only the red fluorescence changed with glyphosate concentrations from 0 to 500 ppm, while the blue fluorescence remained virtually unaffected. The change in red fluorescence could be explained by the interaction between the glyphosate and the surface functional groups of the CDs. This interaction resulted in the quenching of the red fluorescence due to a decrease in radiative emissive pathways from the surface; in contrast, the blue emission was unchanged as this stemmed from the cores of the CDs, which did not interact with the pesticide. Following excitation at 405 nm, the red to blue fluorescence ratio was obtained at every glyphosate concentration using Equation (1).

(1) $${Ratio}_{red\;to\;blue}=\frac{Integrated\;area\;of\;the\;red\;fluorescence\;from\;600-800\;\mathrm{nm}\;(\lambda_{ex}=405\;\mathrm{nm})}{Integrated\;area\;of\;the\;blue\;fluorescence\;from\;415-600\;\mathrm{nm}\;(\lambda_{ex}=405\;\mathrm{nm})}$$

Since the safe maximum concentration of glyphosate for drinking water in Canada is ~0.2 ppm, we determined the sensing capability of the CDs in the low ppm (0–10 ppm) range {Formatting Citation}. As shown in Figure 3, following excitation at 405 nm, the ratio of red to blue fluorescence decreased with the increase in the concentration of glyphosate, where a negative linear response was observed with R^2^ = 0.996. The limit of detection (LOD) of this ratiometric approach was calculated as 0.03 ppm using the standard deviation of the response of the curve (Sy) and the slope of the linear regression (S) following Equation (2):

(2) $$LOD=3.3(S_{y}/S)$$

Next, we determined how the pH affected the spectral properties of the CDs. The same measurements were performed as for water, but with the pH adjusted to 3 using HCl or 10 using NaOH, to mimic the different environmental pH conditions that can occur after natural events such as acid rain and or unnatural events such as chemical leaks in water sources. As shown in Figure 3, the R^2^ = 0.474 and 0.994 for pH 3 and 10, respectively, with an LOD at 0.03 ppm. The lower R^2^ correlation for pH 3 reflects decreased sensitivity to the pesticide. At pH 3, both the glyphosate and the surfaces of the CDs, which comprise similar functional groups, are fully protonated. This results in a decrease in the dipole moment and a reduction in the electrostatic interactions between the glyphosate and the surfaces of the CDs (vide infra).

We also measured the sensing capabilities of the CDs at high ppm concentrations. This was carried out to determine their potential to sense glyphosate in food samples. The regulatory limit for food samples in Canada can range from 0.1 to 400 ppm [39]. The ratio of red to blue fluorescence decreased with increasing glyphosate concentrations at pH 3 (Figure S1A), in water (Figure S1B), and at pH 10 (Figure S1C), where a negative exponential response was observed with R^2^ = 0.948, 0.999, and 0.998 and an LOD of 9.0, 5.0, and 6.0 ppm, respectively. As with the low ppm concentration range for pH 3, a lower R^2^ correlation was also observed at high ppm. Although the correlation was stronger for higher ppm, there was a noticeable decrease in sensing ability, likely due to reduced surface interactions.

Lastly, we determined whether our CDs were specific to glyphosate, by testing them with non-organophosphorus and organophosphorus pesticides. Specifically, we compared their sensing capabilities against glyphosate, boric acid, and Phosmet by measuring changes in the red to blue ratio as described above. As shown in Figure S2, the CDs only responded to glyphosate, with no change in the red to blue ratio for boric acid and Phosmet with increasing concentrations, suggesting that our CDs have strong sensitivity and selectivity for glyphosate.

### 3.3. Dynamic Quenching of the Red Fluorescence

Next, we determined how the interaction between glyphosate and CDs caused fluorescence quenching. Fluorescence quenching is the nonradiative loss of excitation energy from a fluorophore (CDs) through an interaction with a quencher (glyphosate). To determine whether this interaction was static or dynamic, we investigated the change in the area of the red to blue fluorescence ratio as a function of the change in temperature. In the case of dynamic quenching, glyphosate would be expected to interfere with the emission of the excited state of the fluorophore after the formation of an excited state. As the temperature and the kinetic energy of the system increase, more collisions should occur between the surfaces of the dots and the glyphosate. In contrast, static quenching occurs when the initial formation of the emissive excited state is inhibited, preventing any change in fluorescence lifetime with an increased quencher concentration (i.e., glyphosate) [40].

**Figure 4:**
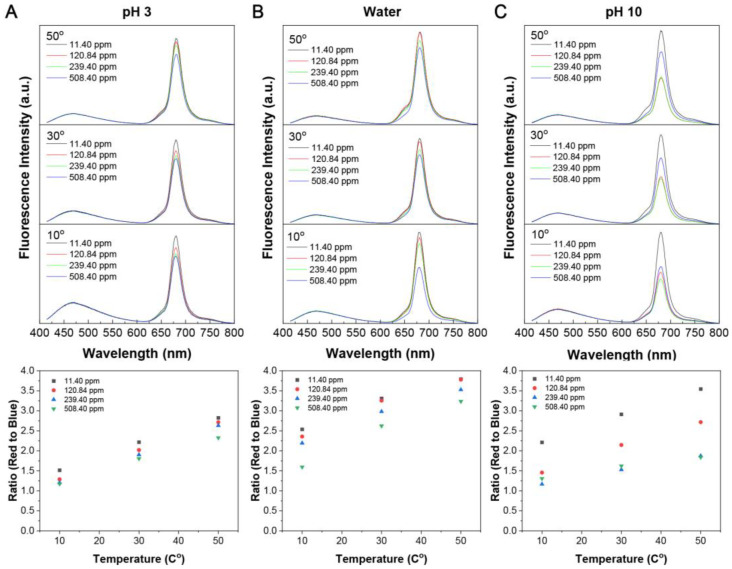
**Dynamic quenching of the CDs by the glyphosate pesticide**. Upon excitation at 405 nm, a change in fluorescence is observed in the red region; the ratio of the integrated area of the red and blue emission peaks shows a negative linear response to the change in glyphosate concentration. The ratio of red to blue fluorescence increases with increasing temperature. (**A**) pH 3, (**B**) water, (**C**) pH 10.

As shown in Figure 4, the ratio of red to blue fluorescence increased with temperature. This effect was observed at pH 3 (Figure 4A), in water (Figure 4B), and at pH 10 (Figure 4C), which can be explained by the increase in collisional quenching between the CDs and the glyphosate with the increase in temperature. These findings suggest that the nature of the interaction is most likely ionic rather than through hydrogen bonding [41,42]. To further support our findings, a Stern–Volmer analysis plot was utilized to obtain more quantitative data on the fluorescence quenching process. As shown in Figure S3, interactions between glyphosate and the CDs supported collisional/complexional quenching from 0 to 200 ppm with a linear relationship, thus confirming the dynamic quenching hypothesis. Moreover, as the concentration of glyphosate increased, a downward curve was observed, suggesting limited access to fluorophores (point of saturation). This could be circumvented by increasing the concentration of CDs in the solution [40,41,42]. As shown in Figure S4, an additional Stern–Volmer analysis was performed to further elucidate the quenching mechanism and the effect of temperature. All three slopes remained the same, with a positive linear relationship, suggesting that dynamic quenching occurred. Lastly, as previously mentioned, the fluorescence lifetimes would remain unchanged in a static quenching paradigm, and we investigated the effect of an increase in glyphosate on the blue and red fluorescence lifetimes. In Figure S5, we can see a decrease in the fluorescence lifetimes for both blue and red emissions, further supporting that the interaction of CDs with glyphosate is purely dynamic.

### 3.4. Ionic Interactions of CDs with Glyphosate

**Figure 5:**
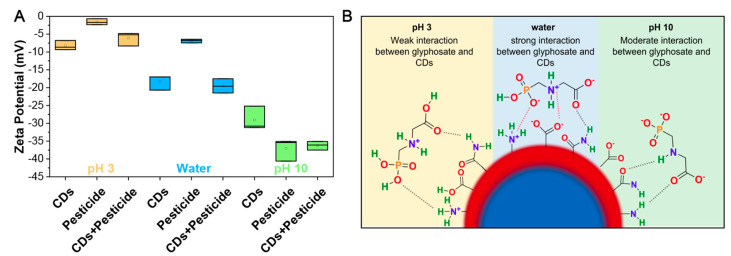
**The surface charge of CDs supports different interactions with glyphosate.** (**A**) A graph shows the change in zeta-potential (mV) for 10 mg/mL of dispersed CDs, 120 ppm of glyphosate, and a mixture of both in water alone (blue, middle) or at pH 3 (yellow, left) or pH 10 (green, right). (**B**) A schematic shows the interaction of glyphosate pesticide with the different functional groups present at the surfaces of the CDs at pH 3, in water (pH7), and at pH 10. Following the protonation and deprotonation events, weak, strong, and moderate interactions are predicted to occur. The black and red dashed lines represent the hydrogen bonding and the ionic bonding, respectively.

To reveal how glyphosate interacted with the surfaces of the CDs, we followed the change in surface charge using zeta-potential measurements. As shown in Figure 5A, as the pH increased, there was a considerable decrease in the surface charge of the CDs, glyphosate (pesticide), and mixture of both. This decrease can be explained by the protonation and deprotonation of the functional groups occurring at the different pH values.

Both CDs and glyphosate comprise similar functional groups, namely carboxyls and amines, which have pKa values in the ranges of 4.0–5.0 and 9.5–11.0, respectively. Glyphosate also contains a phosphate group with three pKa values at 2.1, 7.2, and 12.4. At pH 3, the functional groups of both CDs and glyphosate should be fully protonated, which supports their measured zeta-potential values of −8 mV and −2 mV, respectively. In this case, few ionic interactions occur, and we hypothesize that interactions between the CDs and glyphosate are mediated mostly through hydrogen bonding. Hydrogen bonds are weaker than their ionic counterparts and this could explain why a decrease in sensitivity is observed at pH 3 (R^2^ = 0.47427 at low ppm; R^2^ = 0.9474 at high ppm). In water, the amine groups present at the surfaces of the CDs should be fully protonated, whilst the carboxyl groups would be deprotonated, supporting the decrease in zeta potential to −18 mV. Glyphosate in water should experience the same protonation and deprotonation events as CDs, with the exception that the phosphate group will be partially deprotonated to give a surface charge of −7 mV. The difference in surface charge between the CDs and glyphosate would mediate ionic interactions. This is supported by the sensing ability of CDs in water at both low and high concentrations of glyphosate, with respective R^2^ correlations of 0.9998 and 0.9962, respectively. Lastly, at pH 10, both functional groups of CDs would be deprotonated, causing the measured surface charge to drop to −29 mV. The glyphosate at pH 10 would similarly experience full deprotonation at all three functional groups (i.e., amine, carboxyl, and phosphate), causing a measured surface charge of −37 mV. Although the difference in surface charge is almost equivalent to the measurements in water, the highly negatively charged surfaces of CDs and glyphosate would cause a moderate vs. strong ionic interaction when compared to water (R^2^ = 0.9943 at low ppm; R^2^ = 0.9975 at high ppm). 

## 4. Conclusions

In summary, we describe the synthesis and characterization of a novel CD nanosensor from L-glutathione and formamide precursors. We demonstrate a proof of concept for the ratiometric sensing of the glyphosate pesticide in aqueous dispersions at different pH levels and temperatures. This self-referencing approach achieved LODs as low as 0.03 ppm over different concentrations. Thus, this sensitive nanosensing tool could be used to detect glyphosate in water samples as well as food, where LODs are needed at increased ppm levels. With their facile synthesis and dual fluorescent properties that allow for precise ratiometric measurements, these CDs could be suitable as new glyphosate nanosensors, offering great advantages over more costly particles that require more specialized methodologies and infrastructure. Lastly, we show that these CDs do not detect pesticides such as boric acid and Phosmet, suggesting that they have high selectivity and sensitivity for glyphosate. Since the interaction between CDs and substrates can change with surface modifications and functionalization, other CDs could be generated for the ratiometric sensing of other contaminants, including heavy metals, pharmaceutical drugs, and biological agents.

## Figures and Tables

**Table 1:** Analytical parameters of some sensing methods used for glyphosate detection.

Pesticide	Analytical Methods	Detection Limit	References
**Glyphosate**	High-performance chromatography (HPLC)	50 ng/mL (0.05 ppm)	[21]
	Gas chromatography–mass spectrometry (GC-MS)	0.1 ug/mL (0.0001 ppm)	[22]
	Capillary electrophoresis (CE)	85 ng/mL (0.085 ppm)	[23]
	Inductively coupled plasma mass spectrometry (ICP-MS)	0.7 ug/mL (0.7 ppm)	[24]
	Carbon dots	0.4 ng/mL (0.0004 ppm) 0.8 ng/mL (0.0008 ppm) 9 ng/mL (0.009 ppm) 12 ng/mL (0.012 ppm) 0.6 umol/L (0.10 ppm)	[25] [26] [27] [28] [29]

## Data Availability

Data are contained within the article or supplementary material. Any additional data concerning the work in this study are available on request from the corresponding author.

## Supplementary Materials

The following supporting information can be downloaded at: https://www.mdpi.com/article/10.3390/s23115200/s1. Figure S1. High concentration of glyphosate sensing based on ratiometric measurements. Upon excitation at 405 nm, a change in fluorescence is observed in the red region; the ratio of the integrated area of the red and blue emission peak shows a negative exponential response to the change in glyphosate concentration: (A) pH 3 with LOD = 0.03 ppm; (B) water with LOD = 0.03 ppm; (C) pH 10 with LOD = 0.03 ppm. Figure S2. Bar graph comparing the fluorescence responses of the CDs to different concentrations of pesticides. CDs only show selectivity and sensitivity to glyphosate. Figure S3. Stern–Volmer plot at different environmental pH. A linear response can be observed from 0 to 200 ppm (red line) and a non-linear curved line response for concentrations of 200–500ppm. (A) At pH 3; (B) water; (C) at pH 10. Figure S4. Stern–Volmer plot. A positive linear response can be observed from 0 to 500 ppm of glyphosate in water at 10 °C, 30 °C, and 50 °C. Figure S5. Blue and red fluorescence lifetime analysis. We observe a decrease in both lifetimes at pH 3, in water, and at pH 10, indicating dynamic quenching.
