# Supplementary material for: Ratiometric Sensing of Glyphosate in Water Using Dual Fluorescent Carbon Dots
Source: Sensors (Basel). 2023 May 30;23(11):5200. doi: 10.3390/s23115200 (PMC10255972; doi:10.3390/s23115200)
Supplement: Supplementary file 1 [file sensors-23-05200-s001.zip › sensors-2297160-supplementary.pdf]

Supplementary Information

Ratiometric sensing of glyphosate in water using dual fluorescent carbon dots

Adryanne Clermont-Paquette[1,2,3], Diego-Andrés Mendoza[1,2], Amir Sadeghi[1,2], Alisa Piekny[3], Rafik Naccache[1,2,*]

[1] *Department of Chemistry and Biochemistry, and the Center for NanoScience Research, Concordia University, Montreal, QC, Canada, H4B 1R6*

[2] *Quebec Centre for Advanced Materials, Department of Chemistry and Biochemistry, Concordia University, Montreal, QC, Canada, H4B 1R6*

[3] *Department of Biology, and the Centre for Microscopy and Cellular Imaging, Concordia University, Montreal, QC, Canada, H4B 1R6*

* = Corresponding author (email: rafik.naccache@concordia.ca)

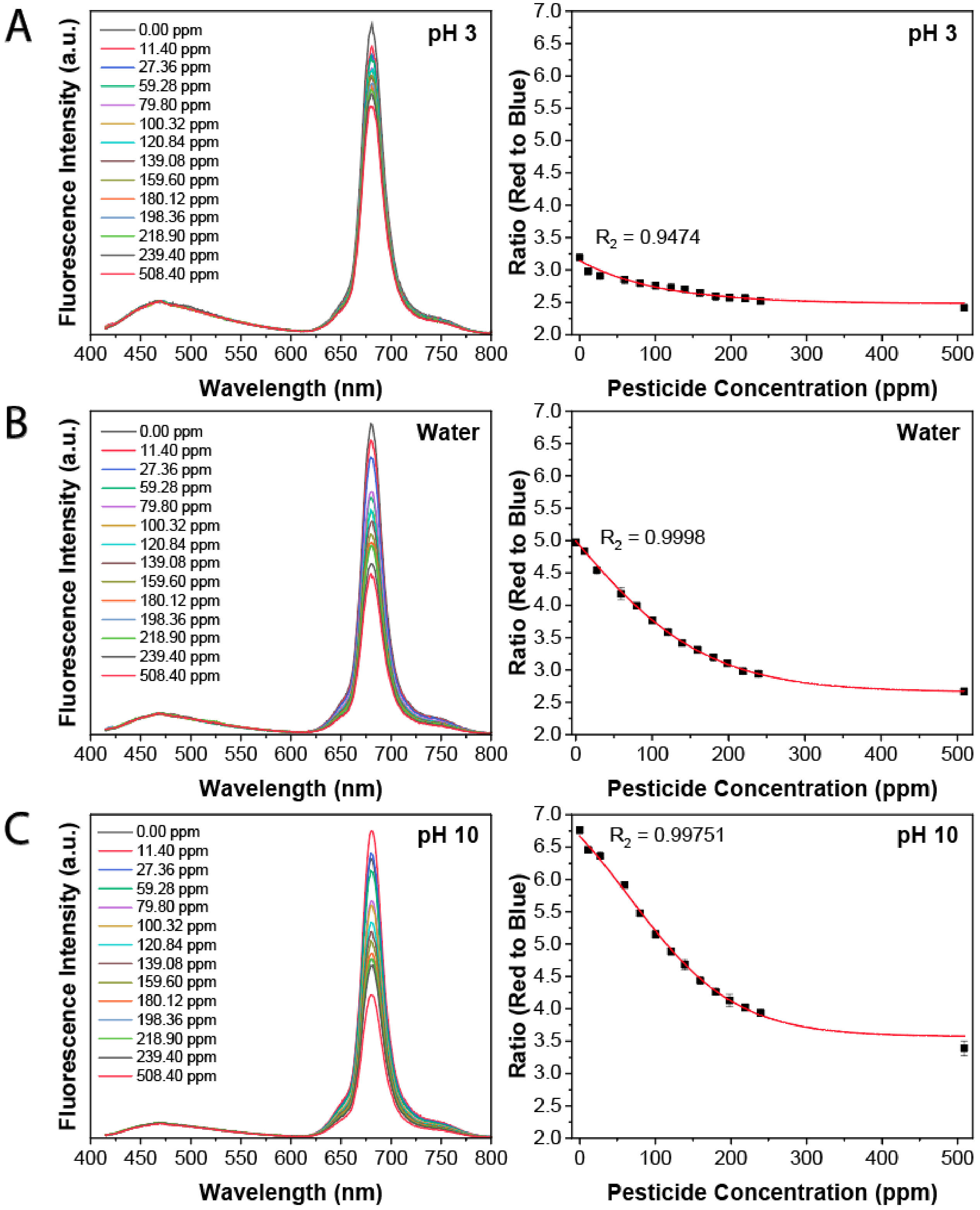

Figure S1. **Dual emissive CDs ratiometrically sense high concentrations of glyphosate.** Graphs on the left show the change in fluorescence intensity for different wavelengths (nm) and concentrations of glyphosate at different pH; (A) pH 3, (B), water, (C), pH 10. Graphs on the right show the ratio of red to blue fluorescence for different glyphosate concentrations for each condition, with the linear regressions (R^2) as indicated.

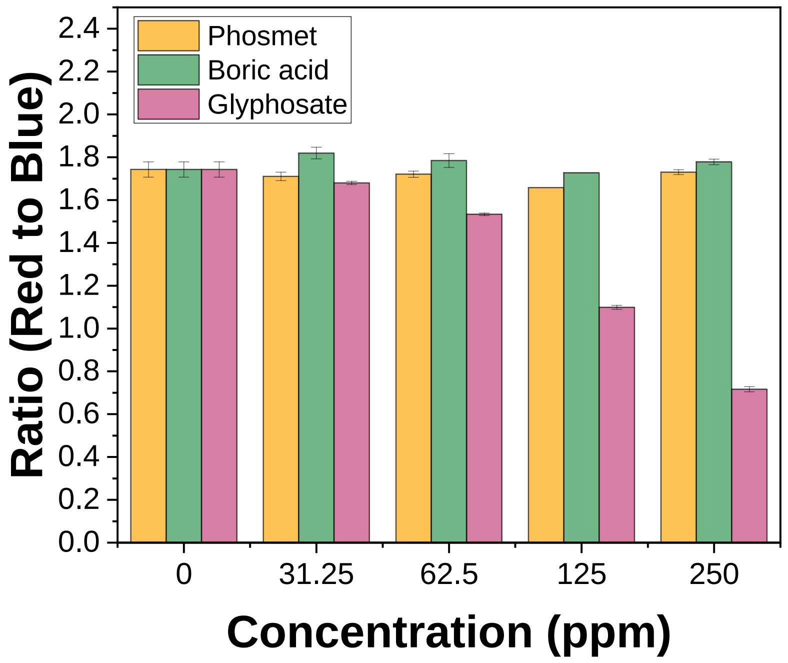

Figure S2. **Dual emissive CDs are only sensitive to glyphosate.** A bar graph shows the ratio of red to blue fluorescence of the CDs after treatment with different concentrations of pesticides in ppm (yellow, Phosmet; green, Boric acid; pink, Glyphosate).

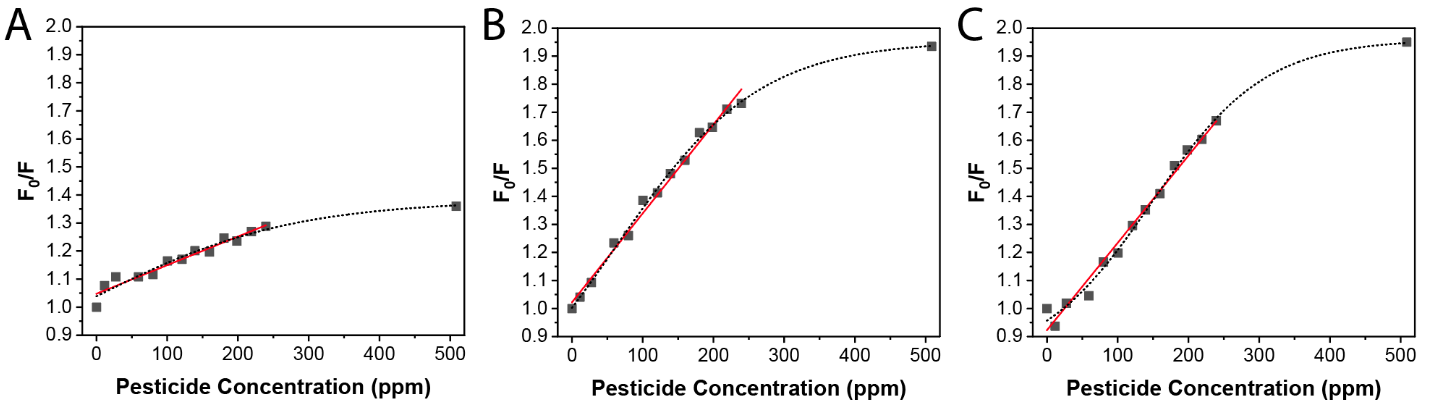

Figure. S3. Stern-Volmer Plots show linear correlation of glyphosate sensing at different pH. Graphs show measurements of the fluorescence quenching at varying concentrations of the quencher (glyphosate) in ppm. (A) at pH 3; (B) Water; (C) at pH 10. The red line shows the positive linear response.

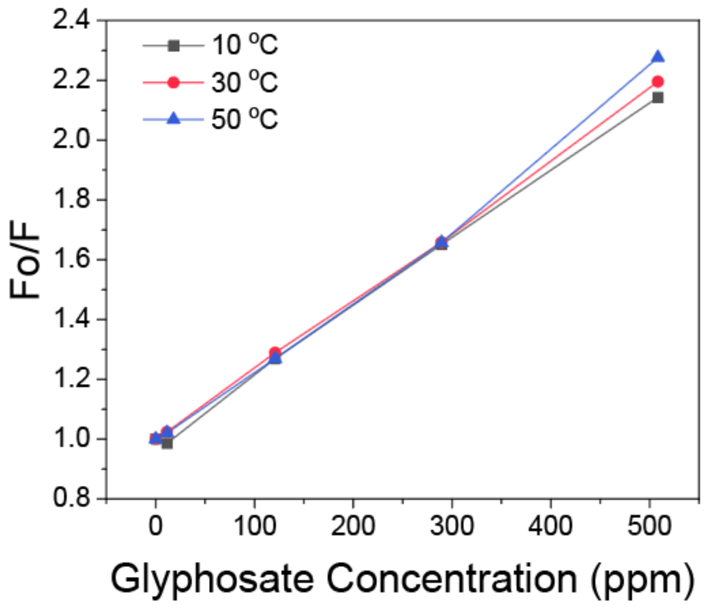

Figure S4. A Stern-Volmer Plot shows linear correlation with glyphosate sensing at different temperatures. A graph shows measurements of the fluorescence quenching at varying concentrations of the quencher (glyphosate) in ppm, in water at 10 ºC, 30 ºC, and 50 ºC.

Glyphosate Concentration	Lifetime at pH3	Lifetime in Water	Lifetime At pH10
11.40 ppm	4.714 ns	5.368 ns	5.017 ns
508.40 ppm	3.323 ns	4.110 ns	3.929 ns

Glyphosate Concentration	Lifetime at pH3	Lifetime in Water	Lifetime At pH10
11.40 ppm	0.2625 ns	0.2147 ns	0.2534 ns
508.40 ppm	0.1130 ns	0.2056 ns	0.1779 ns

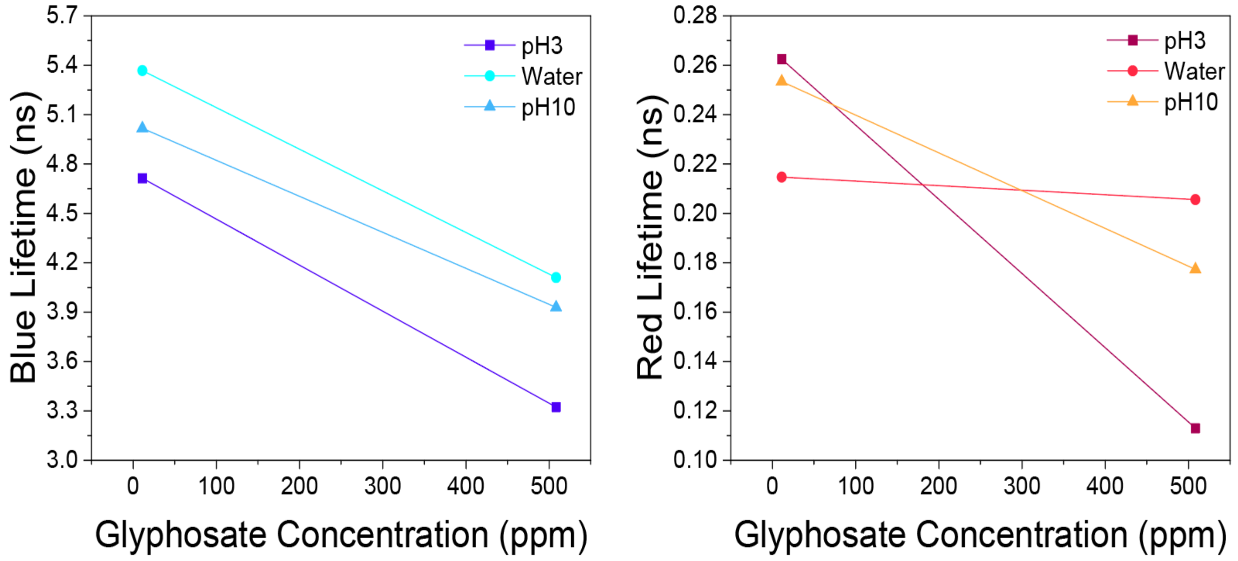

Figure S5. Blue and red fluorescence lifetime analysis shows dynamic quenching with glyphosate. Graphs show the fluorescence lifetimes of both blue (left) and red (right) spectra from the CDs after treatment with varying concentrations of glyphosate and in different pH (purple or maroon, PH 3; aqua or red, water; blue or yellow, pH 10). The average lifetimes for specific concentrations are shown in the tables above.
